# Prolonged Viability of Senecavirus A in Exposed House Flies (*Musca domestica*)

**DOI:** 10.3390/v14010127

**Published:** 2022-01-11

**Authors:** Justin Heath Turner, Willian Pinto Paim, Mayara Fernanda Maggioli, Cristina Mendes Peter, Robert Miknis, Justin Talley, Fernando Vicosa Bauermann

**Affiliations:** 1 Department of Veterinary Pathobiology, College of Veterinary Medicine, Oklahoma State University (OSU), Stillwater, OK 74078, USA; justin.turner11@okstate.edu (J.H.T.); ppaimw@gmail.com (W.P.P.); mayara.maggioli@okstate.edu (M.F.M.); cristina.mendes_peter@okstate.edu (C.M.P.); 2 Department of Entomology & Plant Pathology, Ferguson College of Agriculture, Oklahoma State University (OSU), Stillwater, OK 74078, USA; 3 Laboratório de Virologia, Faculdade de Veterinária, Universidade Federal do Rio Grande do Sul, Porto Alegre 90040-060, Brazil; 4 United States Department of Agriculture Animal and Plant Inspection Service Veterinary Service Strategy and Policy Office of Interagency Coordination, Fort Collins, CO 80526, USA; robert.a.miknis@usda.gov

**Keywords:** insect, house flies, mechanical vector, *Picornaviridae*, *Poxviridae*, virus tenacity

## Abstract

House flies (*Musca domestica*) are often present in swine farms worldwide. These flies utilize animal secretions and waste as a food source. House flies may harbor and transport microbes and pathogens acting as mechanical vectors for diseases. Senecavirus A (SVA) infection in pigs occurs via oronasal route, and animals shed high virus titers to the environment. Additionally, SVA possesses increased environmental resistance. Due to these reasons, we investigated the tenacity of SVA in house flies. Five groups of flies, each composed of ten females and ten males, were exposed to SVA, titer of 10^9.3^ tissue culture infectious dose (TCID_50_/mL). Groups of male and female flies were collected at 0, 6, 12, 24, and 48 h post-exposure. For comparison purposes, groups of flies were exposed to Swinepox virus (SwPV). Infectious SVA was identified in all tested groups. Successful isolation of SVA demonstrated the titers varied between 10^6.8^ and 10^2.8^ TCID_50_/mL in female groups and varied from 10^5.85^ to 10^3.8^ TCID_50_/mL in male groups. In contrast, infectious SwPV was only detected in the female group at 6 h. The significant SVA infectious titer for prolonged periods of time, up to 48 h, indicates a potential role of flies in SVA transmission.

## 1. Introduction

Senecavirus A (SVA) is a nonenveloped virus member of the *Picornaviridae* family within the *Senecavirus* genus. The single-stranded positive-sense RNA genome is about 7.2 kb long and contains a single open reading frame [1]. SVA was identified and characterized in 2002 as a cell culture contaminant and later was occasionally detected in isolated cases of idiopathic vesicular disease in pigs [1,2,3]. However, in 2015 there was an increased number of vesicular disease (VD) cases associated with the presence of SVA in the US [4]. In 2015, similar cases were also described in Brazil, and reports emerged from many important pork-producing countries in Asia and South America [5,6,7,8].

The VD clinical presentation associated with SVA is indistinguishable from that of other viral pathogens associated with VD in swine, including swine vesicular disease, vesicular stomatitis, vesicular exanthema of swine, and foot-and-mouth disease [9]. Pigs are usually infected via the oronasal route, and after a short incubation period of typically less than five days, animals start displaying lethargy and lameness followed by the development of vesicles [9,10]. These lesions are usually present in the snout, oral mucosa, and/or feet. Infected animals shed a significant amount of viruses through vesicular fluid, feces, nasal, and oral secretions [11,12]. SVA infection in pigs is usually self-limited, and most animals are clinically recovered in about two weeks. However, the virus persists in the tonsils and reactivation is possible [13].

House flies (*Musca domestica*) commonly feed in animal secretion and excretion. This feeding behavior provides the opportunity for the flies to interact with contaminated material and transport pathogens harbored on their mouthparts or other body parts. House flies are associated with the potential to transmit bacterial and viral pathogens, including methicillin-resistant *Staphylococcus aureus* and African swine fever virus [14,15,16].

Despite the importance of SVA, many aspects of the epidemiology and ecology of this virus remain unclear [17,18]. Restricted evaluation of SVA in wildlife and insects identified infectious SVA in mice, and SVA RNA was detected in house flies, and Culicoides [17,18]. However, no viable virus could be isolated from Dipteran members [17,18]. Additionally, there is a paucity of information about virus viability in exposed house flies. In the current study, the viability of SVA in exposed house flies was evaluated. For comparison purposes, the viability of another environment-resistant virus that causes skin disease in pigs, Swinepox virus (SwPV), was also evaluated.

## 2. Materials and Methods

### 2.1. Viruses and Cells

The SVA strain HI/2012-NADC40 kindly provided by Drs. Lager and Buckley, USDA ARS, and the SwPV strain NVSL (USDA-NVSL catalog number 002-PDV) were amplified in swine testis (ST) cells. The cells were cultured at 37 °C with 5% CO_2_ in minimal essential media (MEM) (Corning-Mediatech, Inc., Manassas, VA, USA) supplemented with 10% fetal bovine serum (Avantor-Seradigm, Radnor, PA, USA.), 1% antibiotic–antimycotic 100X (Life Technologies-Gibco, Grand Island, NY, USA), 2 mM l-glutamine (Corning-Mediatech, Inc., Manassas, VA, USA), and 50 μg/mL of gentamicin (Corning-Mediatech, Inc., Manassas, VA, USA). The SVA titer used in the study was 10^9.3^ tissue culture infectious dose (TCID_50_/mL), whereas SwPV titer was 10^6.8^ TCID_50_/mL. The virus titration was conducted using the Reed–Muench method (Reed and Muench, 1938).

### 2.2. House Flies

House flies (*Musca domestica*) were obtained from the colony at Oklahoma State University. The colony was started by combining lab-raised flies from the USDA-ARS Manhattan, Kansas, and wild-caught flies from Stillwater, Oklahoma. The used flies were at least ten generations apart from the initial population. Flies were housed at 80% humidity and 26.7 °C. The larvae were raised on a mixture of wheat bran, calf manna, and vermiculite.

### 2.3. Study Design

**Figure 1:**
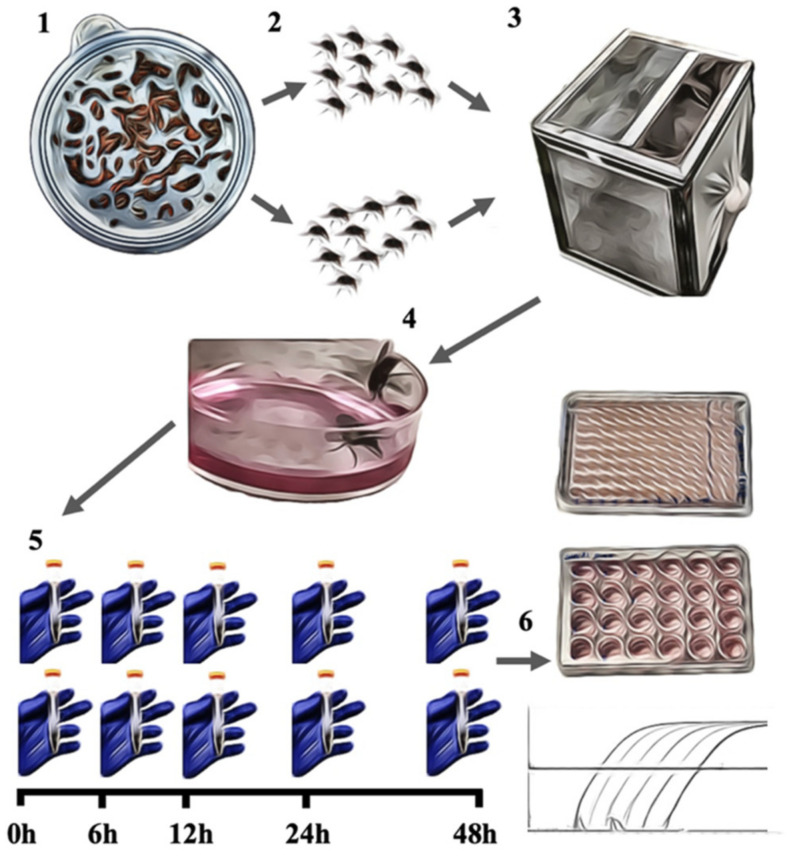
Study design representation. (1) Fly larvae were cultivated; (2) newly emerged flies were sexed; (3) a total of 10 female and 10 male flies were placed in each cage; (4) flies were then exposed to SVA or SwPV solution; (5) flies were collected at five time points and subgroups of five flies from both genders were processed in PBS or in DNA/RNA Shield; (6) the samples were then processed for virus isolation, virus titration, and quantification of the viral nucleic acid.

For viral exposure, newly emerged and unfed house flies were sexed and divided into five groups for each virus (Figure 1). Each group was composed of 10 female and 10 male flies, which were kept in a cage with a 30.5 cm^3^ volume. The cages were sided by an aluminum screen and had a mesh stockinette sleeve. Two milliliters of virus solution was placed in a petri dish with a diameter of 25 mm, which was placed inside each cage. After 60 min, the virus solution was removed, and water and sugar cubes were placed in the cages through the sleeve. Cages were kept in a BSL2 area in a room with a temperature ranging from 21 to 24 °C. Flies from one cage were collected at 0, 6, 12, 24, and 48 h post-exposure in each sampling point. At collection, the cage was kept at −20 °C for at least 10 min. The flies were then collected and sexed, and males and females were separated and stored in 15 mL conical tubes. Flies were kept in a −80 °C freezer for later processing. Nonexposed flies were used as a negative control. At the time of processing, flies were further divided into two groups. Five female and five male flies were placed in 500 µL of DNA/RNA Shield (Zymo Research, Irvine, CA, USA), each group in separate tubes. Similarly, the remaining flies were placed in tubes with 500 µL of PBS (Corning-Mediatech, Inc., Manassas, VA, USA). The flies in PBS and DNA/RNA Shield were crushed with a pestle, and the homogenate was transferred to 0.3 mm ZR BashingBead Lysis Tubes (Zymo Research, Irvine, CA, USA) and vortexed twice at 2000 rpm for 2 min in a plate shaker. Samples were then centrifuged at 5000× *g* for 10 min, and the supernatant was used for testing.

### 2.4. Virus Isolation and Titration

The supernatant of flies prepared in PBS was used for virus isolation (VI) and titration. VI was conducted in 24-well plates with 70% confluent ST cells. A total of 100 µL of the supernatant was allowed to adsorb for 1 h. The inoculum was then removed from each well and washed 3 times with 500 µL of MEM. After washing, 500 µL of MEM was added to each well and the plate was incubated for 48 to 96 h. A total of three blind passages were conducted. At the end of the third passage, cells were examined for cytopathic effect using an inverted light microscope. The fly homogenates (original inoculum) of the positive VI samples were subsequently submitted to the virus titration assay using the limiting dilution method, and the titer was determined as previously described [19]. Viral titrations were conducted in triplicates.

### 2.5. Nucleic Acid Purification and Amplification

The nucleic acid from fly homogenates (both PBS and DNA/RNA Shield processed groups) and the product of the freeze and thaw of the third passage of the VI assay was extracted using the Quick-DNA/RNA Viral kit (Zymo Research) following the manufacturer’s protocol. Additionally, nonexposed flies were prepared as negative controls. SVA purified RNA and SwPV DNA were used as positive controls. The SVA RNA was then amplified using the Luna Universal Probe One-step RT-qPCR Master Mix, while the SwPV DNA was amplified using Luna Universal Probe qPCR Master Mix (New England Biolabs, Ipswich, MA, USA). The reactions were prepared following the manufacturer’s recommendations. Briefly, 2 µL of extracted viral nucleic acid was added to the master mix. The SVA primers were the 5′-GTAGCCAAGAGGGTTCAAGATT-3′ and 5′- CAGTAGACTTCTCGACCTCCT-3′. The probe was 5′-/56-FAM/CGGATTAGCGGGTCTCCTCACAAA/36-TAMSp/-3′. The SwPV primers were primer 5′-GTCGTCGGTCGCTGTTAAAT-3′ and 5′-TGGTTCACCCGGTAGATAGT-3′. The used probe sequence was 5′-/56-FAM/AACATCGAGGACTTTGCTCCGGAC/36-TAMSp/-3′. All samples were run in an Applied Biosystems 7500 Real-Time PCR System (Life Technologies - Applied Biosystems, Foster City, CA, USA). The amplification cycle was composed of an initial reverse transcription step at 55 °C for 10 min for SVA samples. For all the SVA and SwPV samples, the remaining steps included 1 cycle at 95 °C for 60 s, followed by 40 cycles at 95 °C for 15 s, and 60 °C for 30 s. Both the samples and controls were run in duplicates.

## 3. Results and Discussion

Picornaviruses are usually highly transmissible, with rapid replication, and high titers are shed [13,20,21]. Despite the importance of SVA, many aspects of its ecology and the possible role of insects in mechanically transmitting SVA remain unknown [17,18]. Typically, many flies are found in swine farms worldwide, and house flies feed on microbial-rich substrates, animal secretion, feces, and decomposing carcasses and tissues [22]. Although SVA RNA was identified in house flies and *Culicoides*, no viable SVA was detected in either of these Dipteran members [17,18]. Previous studies primarily investigated the potential spread of pathogens associated with human diseases and have shown that house flies can carry pathogens from one area to another [15,23,24].

**Figure 2:**
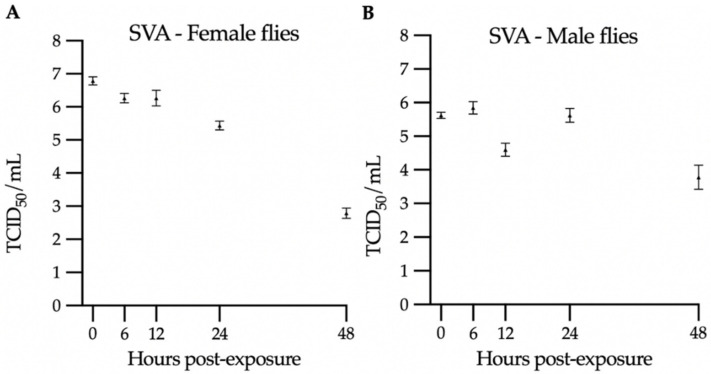
Virus titration in SVA-exposed flies: (**A**) female groups and (**B**) male groups. The triangles represent the average of the triplicate titration, and the bars represent the standard error of the mean.

Here, house flies were exposed to SVA and SwPV to evaluate the length of virus viability. SVA and SwPV nucleic acids were successfully recovered in all collection time points in both female and male flies (Table 1). Remarkably, high levels of infectious SVA were identified even at 48 h after fly exposure (Figure 2). On the other hand, infectious SwPV was limited to one group. The differences in the viability of both SVA and SwPV in exposed flies are remarkable. Both viruses are known to be highly resistant under environmental conditions [12,25,26]. Whereas the study was not designed to identify whether the infectious virus was retrieved from the internal or external parts of the flies, one hypothesis for the prolonged SVA viability compared to SwPV is that SVA resisted the enzymatic digestion process in the flies.

In the SwPV-exposed flies, viable virus was identified only in the female group collected at 6 h post-exposure, and virus titration revealed that the titer was below the assay’s detection limit, lower than 10^1.05^ TCID_50_/mL. While identifying viable virus after 6 h may suggest a potential for transmission, the low levels of the detected virus may indicate that SwPV transmission via flies may have limited relevance to the SwPV ecology.

Contrasting with the SwPV results, high SVA titers were retrieved, and infectious virus was consistently found in all groups. Although the virus concentration in each exposed fly cannot be determined and was likely variable among flies, these results indicate that a significant contamination occurred. The titer for female flies varied from 10^6.8^ to 10^2.8^ TCID_50_/mL, while it varied from 10^5.85^ to 10^3.8^ TCID_50_/mL in male groups. There was a titer decrease trend over time, and therefore there is no evidence of virus replication on the exposed flies based on these results. It was previously described that female house flies harbored higher levels of bacteria than male flies [27]. Here, there was a trend of initial higher titer in female flies, whereas viability trended longer in male flies.

The flies in the current study were exposed to an SVA solution with a titer of 10^9.3^ TICD_50_/mL, and previous studies investigating the pathogenesis of SVA in pigs have shown that shedding in secretions and feces may reach over 10^7^ copies [13,20,21]. Additionally, vesicular lesion tissue and vesicular fluid from SVA-infected pigs often present viral titers comparable to the inoculum used in the present study [20]. Therefore, it is plausible that flies may be infected with comparable viral titers during feeding in naturally infected pig secretion, feces, or tissues. Whereas the SVA minimal infectious dose is unknown, a previous study demonstrated that pigs fed for three consecutive days with feed spiked with SVA containing doses as low as 10^5.0^ TCID_50_/mL had RNA present in the feces and tonsils, and most of the exposed pigs also had viremia [12].

The prolonged viability of SVA in house flies may provide an opportunity for pathogen transfer over some distances due to the house fly flight range. Flies can reach a speed of about 8 km/h, and a previous study collected flies over a three-day period and captured flies released 20 km away [28]. Despite this, flies tend to stay within a smaller area if food sources are available. Significant movement is likely due to environmental circumstances (extreme weather events), unintended transport during animal transportation, or transport in personnel and visitor vehicles.

In the current study, flies were exposed to the virus by naturally interacting and feeding in the virus solution. Therefore, we can conclude that at least one fly in each group was exposed. The different levels of interaction during exposure may explain the variation among groups. Of note, flies were exposed to viruses for a limited time of 60 min and fly contamination was dependent on the insect interaction with the viral solution. It is plausible that flies would be continuously exposed to contaminated material in farms affected by SVA, with a greater potential for contamination.

The results from our study suggest that flies feeding in SVA-contaminated substrate may carry significant infectious SVA for at least 48 h and may play a role in disease transmission in and between farms. Therefore, fly control may be advised to limit virus spread within a farm and to neighboring farms.

## Figures and Tables

**Table 1:** Nucleic acid quantification of SVA and SwPV in exposed flies collected at 0, 6, 12, 24, and 48 h post-exposure. Results are presented as the average of the cycle of quantification (Cq). The duplicate testing was conducted in samples collected in DNA/RNA Shield and before and after virus isolation (Cq pre VI and Cq post VI) in samples collected in PBS.

Virus	Group	Hours Post-Exposure	DNA/RNA Shield Processed Flies	PBS Processed Flies	
			Cq	Cq Pre VI	Cq Post VI
SVA	Female	0	20.6	19.5	13.3
		6	23.5	22.7	13.5
		12	27.9	21.8	14.1
		24	22.0	22.8	14.3
		48	27.5	33.3	14.2
	Male	0	21.5	22.5	13.2
		6	23.5	23.7	13.2
		12	27.9	26.6	13.2
		24	30.2	23.7	13.5
		48	30.5	28.3	14.6
SwPV	Female	0	25.1	30.0	NA
		6	25.8	31.4	27.2
		12	27.5	32.7	NA
		24	32.3	37.7	NA
		48	29.5	36.4	NA
	Male	0	21.9	29.5	NA
		6	27.5	32.7	NA
		12	25.7	32.2	NA
		24	33.7	37.8	NA
		48	32.6	35.2	NA
